# A Co-word Analysis of Selected Science Education Literature: Identifying Research Trends of Scaffolding in Two Decades (2000–2019)

**DOI:** 10.3389/fpsyg.2022.844425

**Published:** 2022-02-18

**Authors:** Tzu-Chiang Lin, Kai-Yu Tang, Shu-Sheng Lin, Miao-Li Changlai, Ying-Shao Hsu

**Affiliations:** ^1^Center for Liberal Arts, National Kaohsiung University of Science and Technology, Kaohsiung City, Taiwan; ^2^Center for Teacher Education, National Kaohsiung University of Science and Technology, Kaohsiung City, Taiwan; ^3^Department of International Business, Ming Chuan University, Taipei City, Taiwan; ^4^Graduate Institute of Mathematics and Science Education, National Chiayi University, Chiayi City, Taiwan; ^5^Department of Digital Multimedia Design, China University of Technology, Taipei City, Taiwan; ^6^Graduate Institute of Science Education, National Taiwan Normal University, Taipei City, Taiwan; ^7^Institute for Research Excellence in Learning Sciences, National Taiwan Normal University, Taipei City, Taiwan

**Keywords:** co-word analysis, research trends, scaffolding, science education, educational technology

## Abstract

This study aims to identify research trends of scaffolding in the field of science education. To this end, both descriptive analysis and co-word analysis were conducted to examine the selected articles published in the Social Science Citation Index journals from 2000 to 2019. A total of 637 papers were retrieved as research samples through rounds of searching in Web of Science database. Overall, this study reveals a growing trend of science educators' academic publications about scaffolding in the recent two decades. In these sample papers, from 1,487 non-repeated keywords, we extracted 286 author-defined keywords shared by at least two studies as a benchmark dictionary. A series of co-word analyses were then conducted based on the dictionary to reveal the underlying co-occurring relationships of the words in title and abstract of the sample papers. Results showed that “scaffolding,” “support,” and “design” were the top three most frequently used keywords during 2000 and 2019. Visualization of co-word networks in each 5-year period further helps clarify both educators' common research foci and relevant research trends. Derived discussion and potential research directions are also provided.

## Introduction

In the last decades, educators have reached a consensus that scaffolding analogises learners' knowledge building with proper supports. Wood et al. (1976) first identified young children's development progression with learning scaffolds provided by their parents. To accomplish tasks in daily life, young children should be equipped with certain skills and corresponding confidence in themselves. In this progression, learning supports act as scaffolding that guides children's advancement beyond what they may achieve simply on their own. With decreasing aids, usually referred to as fading, children may gradually perform tasks in a more self-directed manner. This provides insight into educational reforms from the perspective of instruction practices in the classroom (Bliss et al., 1996). To facilitate meaningful learning, scaffolding is deemed as learner-centered guidance that affords goal setting, knowledge construction, as well as self-reflection during the learning process (Davis and Linn, 2000; Azevedo et al., 2004).

Scaffolding hence reflects ideas of social constructivism (Vygotsky, 1978; Bliss, 1994) and the zone of proximal development (ZPD) (Rogoff, 1990; Metz, 1995, 1997). The set of existing theories depicts instructional strategies that may overcome the gap between what students learn on their own and what they learn with assistance. In the field of science education, the theories have also been shown to fundamentally align with relevant learning approaches such as argumentation (McNeill et al., 2006; Belland et al., 2011; Noroozi et al., 2018), project-based learning (Land and Zembal-Saul, 2003; Reiser, 2004), problem-based learning (Belland et al., 2011; Kim and Hannafin, 2011), as well as inquiry (Reiser, 2004; Hsu et al., 2015). Science educators have exerted impressive efforts to apply scaffolding in sophisticated learning contexts that highlight students' metacognition, higher order thinking skills, and modeling practice (Fretz et al., 2002; Tang et al., 2016; Toleda and Dubas, 2016; Alrawili et al., 2020). Moreover, the development of educational technology has enabled researchers and teachers to accomplish more expected scaffolds in instructional designs (Quintana et al., 2004; Oh and Jonassen, 2007; Kim and Hannafin, 2011). In sum, in the field of science education, scaffolding is an issue that continues to attract educators' attention. Understanding the relationships interwoven with such issue through systematic reviews of the relevant literature may inform educators of the trends and research spaces.

Lin T. C. et al. (2012) demonstrated a review work on studies that focused on the science education field. Their review examined empirical studies published in Social Science Citation Index (SSCI) journals from 1995 to 2009 to identify research trends regarding scaffolding. Most of the studies focused on improving contexts for science learning. However, an unexpected finding was that in the 15 years, researchers invested only limited efforts in unveiling how science teacher education improved teachers' professional development related to scaffolding. It was also surprising that fading, an essential part of the scaffolding process, was precisely described in <10% of the studies. Researchers thereafter reported similar review works regarding scaffolding that addressed various research domains such as literacy learning (Brownfield and Wilkinson, 2018), computer-based scaffolding in STEM education (Belland et al., 2017) as well as metacognitive scaffolding for online information searching (Zhou and Lam, 2019). With regard to technology-enhanced science education, Wu et al. (2021) reviewed 60 studies to identify research trend of technology-enhanced chemistry learning. Chen et al. (in press) targeted 44 academic publications to analyze and report implications of flipped science learning. The previous review works with content analysis on one hand revealed possible research directions, but on the other hand highlighted potential difficulties in certain research issues. Consecutive trends and alternative analytic techniques may be necessary to expand a more systematic view of development in the research field.

Comparing with content analysis used in previous review studies (Lin T. C. et al., 2012; Brownfield and Wilkinson, 2018; Zhou and Lam, 2019), a co-word network analysis potentially provides complementary views to explore the intellectual structure of keywords used by researchers. It is also noteworthy that the process of manual reviews in the content analysis are time-consuming. Researchers may encounter difficulties when they try to examine the research trends based on an overly large quantity of literature or literature from an overly long period of time. Literature review approach such as content analysis on large-size texts in nature requires imaginable efforts. It is almost impossible to consider information that possibly exists beyond coding framework pre-established by researchers. The bibliometric method may be a satisfactory approach that can unveil complex sets of relationships among a large body of literature. Bibliometric analysis stems from library science, and basically taxonomizes literature with certain characteristics. Such research approach is especially important today as the amount of academic literature has mushroomed in recent years with unimaginable speed. Co-word analysis, a kind of bibliometric method, was hence developed to calculate the co-occurrence counts of selected words in literature (Callon et al., 1991). Recently, researchers in education have adopted the method to explore the research themes in educational research (Huang et al., 2020), STEAM literature (Marín-Marín et al., 2021), and intellectual structure of a SSCI-journal's publications (López-Belmonte et al., 2021). In this study, we adopted a series of co-word network analyses to provide visualized network structure of the scaffolding keywords based on co-occurrence relationships.

Furthermore, using author-defined keywords to represent the focal interest of a selected article is valid in the current analysis as the main idea of validity refers to how accurately a method measures what it is intended to measure (Kelley, 1927). Researchers have suggested that content validity is a key for the valid measure (Rubio et al., 2003). Therefore, this study conducted a co-word network analysis of literature to analyze the structure of keyword co-occurrence to uncover the development trends of empirical studies about scaffolding in science education. We especially focus on the thorough understanding of mutual wording that educators inclined to identify their research focuses, approaches, and findings about scaffolding. The keywords designated by authors are especially deemed as key clues to understand how the research content was defined (Assefa and Rorissa, 2013). A co-word analysis of the status and degree of keyword sharing may represent a precise picture of core research foci in the literature.

The review in this study aimed to identify relationships of high-quality academic articles published in Social Science Citation Index (SSCI) journals within the recent 20 years. This study also aimed at revealing ideas associated with scaffolding. Further interpretations of similarities and dissimilarities that science educators regarded in the contemporary research on scaffolding are, therefore, highlighted. The specific research questions are as follows:

1. What keywords regarding scaffolding were designated in the 2000–2019 SSCI journal articles? What is the trend of these keywords for each 5-year period?
2. What is the visualized pattern of co-word analysis of the research articles regarding scaffolding published in the 2000–2019 SSCI journals? What is the trend of the patterns for each 5-year period?

## Data and Methods

### Data

This study conducted several rounds of topic searches on the Web of Science (WoS) database using the keywords “scaffold” and “scaffolding” coupled with other keywords including science, physics, chemistry, biology, earth science, mathematics, environmental science, medical science, STEM, and STEAM. A total of 1,106 papers published in SSCI journals from 2000 to 2019 were retrieved. A series of relevance checks was conducted to filter out some irrelevant papers for the subsequent analysis. First of all, redundant papers from different queries were excluded (*n* = 401). Next, in line with previous review studies (Lin T. C. et al., 2012), we removed some editorial materials (*n* = 5) and correction data (*n* = 1), and only retained article- and review-type papers in the dataset. Note that some incomplete data and some non-English papers (*n* = 4) were also removed. Last, the keywords used for the topic search were again used to examine the relevance of all remaining papers. Those papers only indexed by the system-defined KeywordPlus (a special category defined by the WoS) were removed (*n* = 57). As a result, a total of 637 papers remained for the subsequent analysis. The sample papers cited in this article are asterisked in the reference list.

### The Identification of Keywords

There are many different standards for the presentation of keywords among various journal publications. For example, some journals only allow authors to select keywords from build-in datasets instead of using author-defined keywords, while some other journals publish papers without keywords. In this present study, we found that the keyword column of over 24.5% (156 out of 637 papers) of the articles was empty. To solve this problem, we present the three-step procedure we adopted to re-index all 637 papers.

### Dictionarizing Co-words

First, we dictionarized the available author-defined keywords as the reference to re-index words presented in title and abstract of the selected articles. All the author-defined keywords from 481 papers were collected. As a result, we found a total of 1,487 non-repeated keywords. This set of keywords represents the focus knowledge of the scaffolding research and was treated as the boundary of the keywords used in the field. Second, the frequency of use of every keyword was counted. Among all 2,365 usage counts, approximately one half (*n* = 1,166, 49.3%) were focused on a total of 286 author-defined keywords that were used by at least two researchers in the field. Third, this set of 286 keywords was deemed as a benchmark dictionary to re-index all of the analyzed papers. This process was performed to construct a standardized set of keywords to characterize all 637 papers. In line with the practice of topic searches in the WoS, we selected the two columns of article title and abstract for the process of dictionarization. This also avoided the potential interference of simply dictionarizing the whole article. A co-word analysis was used to detect whether a paper incorporated any of the abovementioned 286 keywords. As a result, all 637 articles were re-indexed with a range of 2–31 dictionarized keywords. Analysis of the keyword structure which evolved in each period of interest from 2000 to 2019 is further discussed based on the perspective of co-word network analysis.

### Co-word Network Analysis

A co-word analysis assumes that the author-defined keywords in the academic publications are the key description of its research content (Dehdarirad et al., 2014; Huang et al., 2020). In this manner, the links between a large number of co-occurring keywords represent the focal interest of the Research Topic in the field. By definition, two keywords of interest that co-occur within the same paper suggest a certain degree of bibliometric relationship between the topics to which the keywords refer (Cambrosio et al., 1993). For example, keywords A and B co-occur if they are used together in a focal research paper. According to Leydesdorff (1989), however, “a word which occurs only once cannot form a co-word linkage” as it may appear occasionally or be determined by researchers in a single study. In the current review, only the co-occurrence links that outperformed the average strength were considered as influential keywords of scaffolding research.

To calculate the co-occurring relationship among keywords, a co-occurrence was formed. In this study, a total of 286 keywords were listed in both rows and columns, where each cell represents the frequency of co-occurrence counts of the two focal keywords. The more frequently the keywords were co-used by the researchers, the higher likelihood that those two keywords represented similar concepts in the context of scaffolding research. On the other hand, two keywords were considered as dissimilar from each other if they were not used together by field researchers. To further observe the co-occurrence network in the scaffolding research, some networking measures of centrality (e.g., degree centrality measures) were adopted to identify the most influential keyword corpus in the field. After the co-occurrence matrix was obtained, VOSviewer version 1.6.15 was used to visualize the co-occurrence structure of the keyword corpus in each period of research from 2000 to 2019.

## Results and Discussion

Descriptive characteristics and research trends regarding scaffolding of the articles published in SSCI journals were revealed through a series of co-occurring keyword analyses and comparisons. Thereafter, we present the results of co-word network analysis to visualize relationships among the keywords in different periods with a 5-year interval from 2000 to 2019.

### Descriptive Characteristics of the Articles

**Figure 1:**
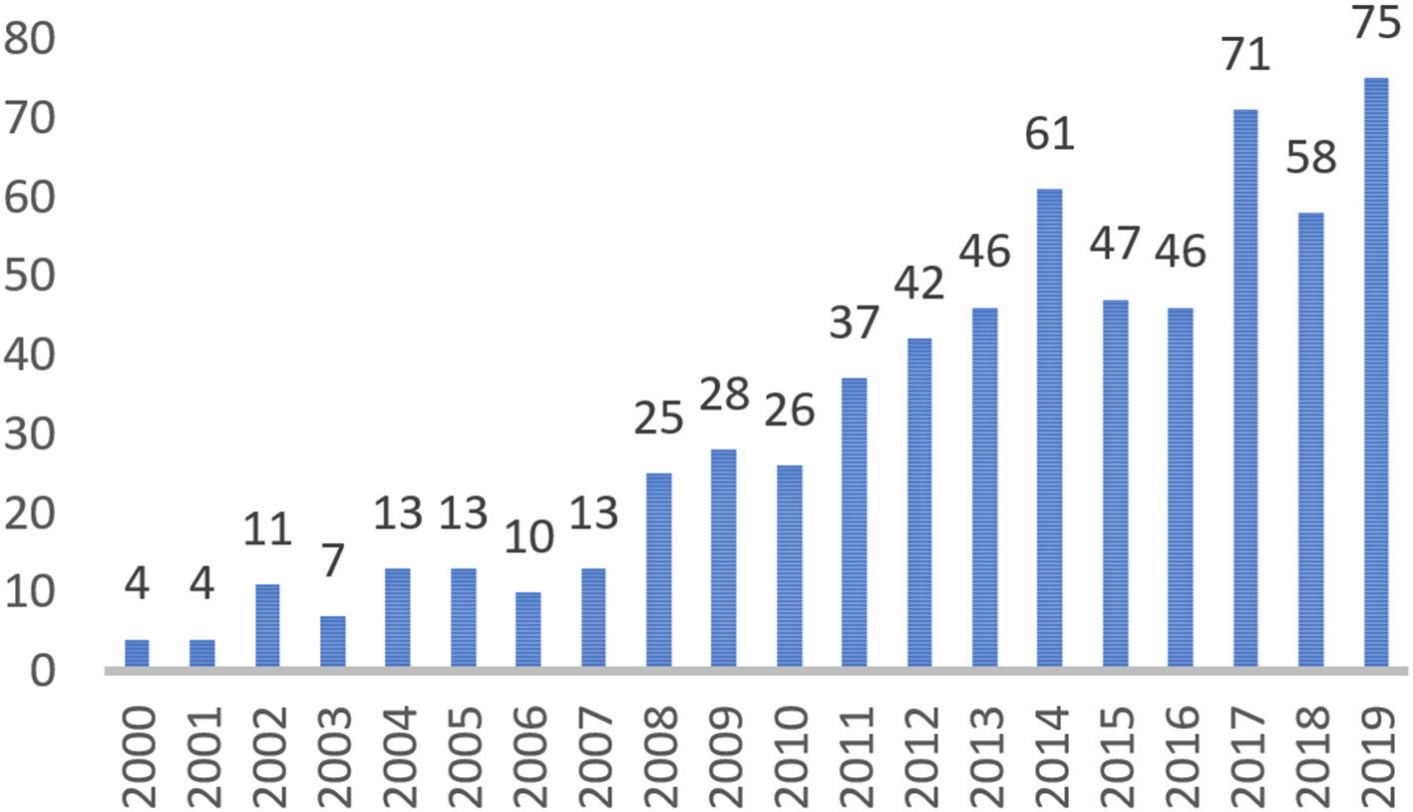
Numbers of the articles on scaffolding from 2000 to 2019.

**Figure 2:**
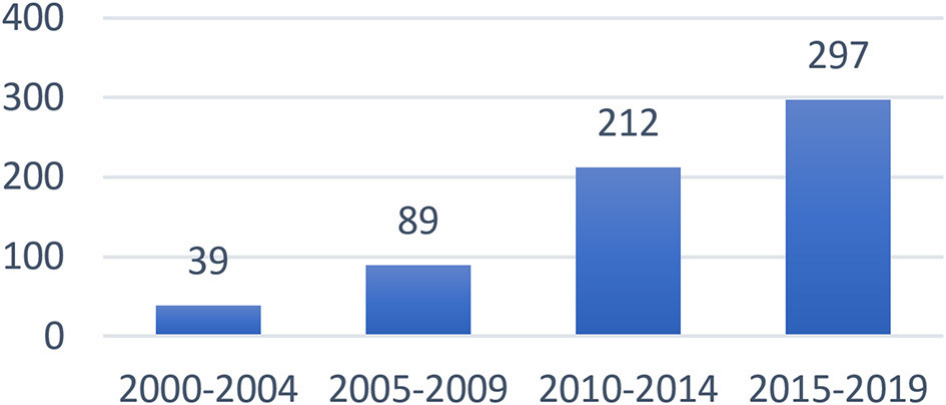
Numbers of the articles on scaffolding in 5-year intervals.

Figure 1 presents the number of articles on scaffolding published in each year from 2000 to 2019. The published articles on scaffolding continued to increase, which indicated that the field was growing and attracting more researchers to contribute their efforts, especially during 2013–2014, 2016–2017, and 2018–2019. The published articles on scaffolding at 5-year intervals also showed an increasing trend. A closer look at the trend analysis indicated an obvious jump from 2005–2009 to 2010–2014 (Figure 2).

**Figure 3:**
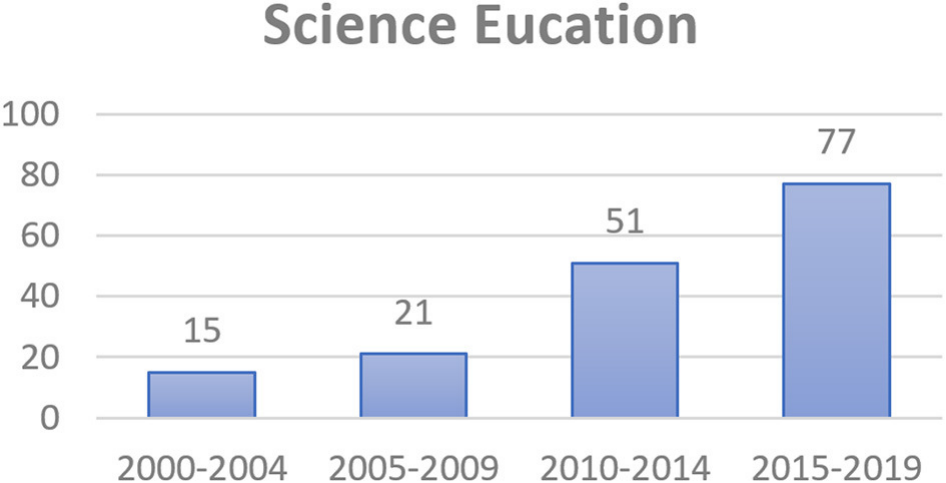
Numbers of the articles published in the science-education journals.

**Figure 4:**
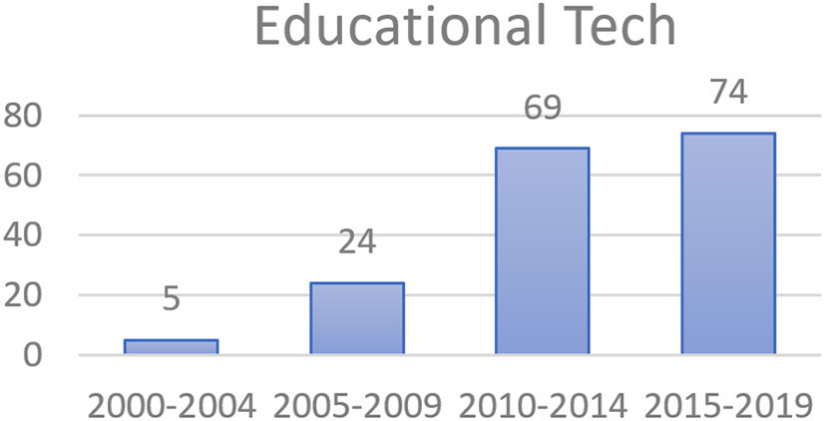
Numbers of the articles published in the educational-technology journals.

Besides, when focusing on science education and educational technology fields, the numbers of selected articles published in the nine journals (Table 1) related to science education and in the 14 journals (Table 2) related to educational technology increased over the 5-year intervals (Figures 3, 4). The trend analysis indicated an obvious jump from 2005–2009 to 2010–2014.

**Table 1:** The selected science-education journal list.

Rank^*^	**Journal title**	**Total cites**	**Impact factor**	**Eigenfactor score**
10	Journal of Research in Science Teaching	6,518	3.870	0.004540
12	Studies in Science Education	718	3.700	0.000590
19	Science Education	5,048	3.500	0.002800
66	Research in Science Education	1,728	2.248	0.001420
100	International Journal of STEM Education	368	1.850	0.000910
133	International Journal of Science and Mathematics Education	1,358	1.578	0.001830
146	International Journal of Science Education	5,613	1.485	0.004160
212	Journal of Baltic Science Education	385	0.915	0.000260
248	Cultural Studies of Science Education	530	0.437	0.000550

* *Nine science-education journals selected from top 250 in the “Education and Educational Research” category of 2019 Journal Citation Report in WoS database*.

**Table 2:** The selected educational-technology journal list.

Rank^*^	**Journal title**	**Total cites**	**Impact factor**	**Eigenfactor score**
3	Internet and Higher Education	3,217	6.566	0.003060
4	Computers & Education	15,521	5.296	0.013370
9	International Journal of Computer-Supported Collaborative Learning	796	4.028	0.000740
27	International Journal of Educational Technology in Higher Education	371	3.080	0.000450
31	British Journal of Educational Technology	4,359	2.951	0.003410
36	IEEE Transactions on Learning Technologies	948	2.714	0.001160
59	International Review of Research in Open and Distributed Learning	2,443	2.297	0.002030
72	Journal of Educational Computing Research	1,475	2.180	0.001030
79	Journal of Computer Assisted Learning	2,352	2.126	0.002050
83	Educational Technology and Society	3,136	2.086	0.003310
95	Australasian Journal of Educational Technology	1,468	1.956	0.000990
96	Interactive Learning Environments	1,197	1.938	0.001940
124	Journal of Science Education and Technology	1,876	1.644	0.001770
191	Research in Science and Technological Education	501	1.111	0.000450

* *14 educational-technology journals selected from top 250 in the “Education and Educational Research” category of 2019 Journal Citation Report in WoS database*.

It should be noted that the jumps occurred in varying sets of trend analyses. The jumps could be because a growing number of researchers valued the importance of scaffolding and explored its effects on learning and teaching after the National Science Council (NRC, USA) released the final Framework for K−12 Science Education in 2011, and the NGSS (Next Generation Science Standards) were published on its website in 2013.

### Trend Analysis of the Keywords and Co-occurring Keywords in the Selected Articles

The results in Table 3 show the top 30 keyword frequencies (about half of the 63 keywords, the frequencies of which were above the average of 23.5) and the derived trends. The total frequency of “scaffolding” is shown as 611 instead of 637 because there were 26 articles which used scaffolding in compound phrases such as computer-based scaffolding (12), distributed scaffolding (4), and metacognitive scaffolding (10). Referring to the trends in Table 3, the highest occurring keywords in the selected articles continued to increase from the first period (2000–2004) to the fourth period (2015–2019). The numbers also indicated a jump from the second period (2005–2009) to the third period (2010–2014). This revealed the same trend with the total number of scaffolding-related articles (Figure 2), the articles published in the journals related to science education (Figure 3), and the articles published in the journals related to educational technology (Figure 4) due to the release of the Framework for K-12 Science Education in 2011 and NGSS in 2013.

**Table 3:** The most frequently used keywords in scaffolding research.

**Rank**	**Keywords**	**2000–2004**	**2005–2009**	**2010–2014**	**2015–2019**	**Total**
1	Scaffolding	**37**	**87**	**207**	**280**	611^*^
2	Support	17	**38**	**106**	**146**	307
3	Design	16	**34**	**87**	**119**	256
4	Knowledge	11	**32**	**60**	**89**	192
5	Instruction	10	31	**56**	**80**	177
6	Context	12	22	**57**	**65**	156
7	Practice	8	25	**46**	**77**	156
8	Cognition	7	23	**49**	**69**	148
9	Teacher	16	14	**47**	**70**	147
10	STEM	7	12	**55**	**67**	141
11	High school	4	20	**41**	**73**	138
12	Science	9	28	**34**	**46**	117
13	Interaction	13	16	**34**	**53**	116
13	Learning environment	7	21	**39**	**49**	116
15	Technology	6	12	29	**46**	93
16	Assessment	4	8	37	**33**	82
17	Experiment	3	9	24	**45**	81
18	Evidence	3	8	25	**42**	78
19	Science education	4	5	31	**36**	76
20	Reasoning	2	13	20	**36**	71
21	Computer	4	7	21	**35**	67
21	Mathematics	4	6	27	30	67
23	Observation	3	11	25	27	66
24	Physics	1	8	26	30	65
25	Modeling	4	10	14	**33**	61
26	Evaluation	6	6	13	**34**	59
27	Case Study	4	6	16	32	58
28	Elementary school	7	7	22	21	57
29	Reflection	3	8	12	32	55
30	Argumentation	5	8	15	26	54
30	Feedback	2	7	23	22	54

* *12 articles in computer-based scaffolding, 4 articles in distributed scaffolding, and 10 articles in metacognitive scaffolding (total = 637).*

*The bold font indicates the occurrence frequency of more than 33 times (one S.D. from the frequency average)*.

For better understanding of the research themes of the selected articles, we classified the keywords into six groups. First are the keywords related to scaffolding characteristics such as support (307), design (256), context (156), learning environment (116), interaction (116), technology (93), computer (67), and feedback (54). Second are the keywords which indicated scaffolding for specific learning performances such as knowledge (192), practice (156), cognition (148), experiment (81), evidence (78), reasoning (71), observation (66), modeling (61), evaluation (59), reflection (55), and argumentation (54). Third are the keywords about teaching (who and when to design and provide scaffolding) such as teachers (147), and in instruction (177) and assessment (82). Fourth are the keywords related to learning disciplines to be learned such as STEM (138), Science (117), mathematics (67), and physics (65). Fifth are the keywords indicating the grades of learners such as high school (138) and elementary school (57), which were two major groups of subjects in the selected studies. Sixth is the most popular methodology used, which was case study (58). A keyword not included in the six groups was science education (76), which is a general keyword used by science educators in their articles.

Even though the trend analysis showed that the three most frequent keywords (scaffolding, support, and design) were the same throughout the entire 20 years, other high-frequency keywords occurred differently in the different 5-year periods. When we selected 33 as the threshold number of high-occurrence keywords, which was one S.D. (9.7) from the frequency average (23.5) of the occurrence frequency, no keywords other than scaffolding occurred (37) in the first period (2000–2004). Regarding keywords that appeared more than 33 times in the second period (2005–2009), only the three previously mentioned most frequent keywords related to scaffolding characteristics were identified, namely scaffolding (87), support (38), and design (34). Regarding an occurrence frequency of more than 33 times in the third period (2010–2014), six keywords related to scaffolding characteristics occurred: scaffolding (207), support (106), design (87), context (57), interaction (34) and learning environment (39); three keywords related to teaching were instruction (56), teacher (47), and assessment (37); three keywords related to learning performance were knowledge (60), cognition (49), and practice (46); and two keywords related to disciplines were STEM (55) and science (34). Regarding an occurrence frequency of more than 33 times in the fourth period (2015–2019), one more keyword (technology) related to scaffolding characteristics occurred in addition to the six keywords in the third period; the same three keywords related to teaching as the third period appeared; five more keywords (experiment, evidence, reasoning, modeling, and evaluation) related to learning performances occurred in addition to the same three high-occurrence keywords as the third period; the same two keywords (STEM and science) related to disciplines as in the third period occurred. Compared to the first three periods, more interest in technology was found together with scaffolding in the fourth period. Moreover, inquiry practices (experiment, evidence, reasoning, and modeling) and higher order thinking skills (reasoning, modeling, and evaluation) seemed to attract attention in the most recent period (2015–2019).

### Comparisons of Co-occurring Keywords in Science-Education Journals and Educational-Technology Journals

Table 4 shows the top 14 frequently co-occurring keywords with co-occurrence times above 23.5 as the average frequency in the selected science education journals. The frequencies of the top co-occurring keywords continued to increase from the first period (2000–2004) to the fourth period (2015–2019). It is worth noting that the trend showed a significant increase from the third period (2010–2014) to the fourth period (2015–2019). Scaffolding hence acted as the most frequently co-occurring keyword during the four periods. When we used 24 (the frequency average was 23.5) as the threshold number of frequently co-occurring keywords, no co-occurring keywords were included in the first period (2000–2004) or in the second period (2005–2009). Regarding 24 or more times of co-occurring frequency in the third period (2010–2014), only two co-occurring keywords were identified, namely scaffolding (51) and science education (26). Compared with the same two co-occurring keywords in the third period, five more co-occurring keywords were identified: support (39) and design (26) related to scaffolding characteristics, teacher (25) related to teaching, and knowledge (24) and practice (19) in the fourth period (2015–2019).

**Table 4:** The frequently co-occurred keywords in the selected science education journals.

**Rank**	**Keywords**	**2000–2004**	**2005–2009**	**2010–2014**	**2015–2019**	**Total**
1	Scaffolding	15	20	**51**	**73**	159
2	Support	6	9	18	**39**	72
3	Science education	6	0	**26**	**32**	64
4	Knowledge	6	10	19	**24**	59
5	Design	5	8	18	**26**	57
6	Teacher	7	8	14	**25**	54
7	Science	5	9	16	19	49
8	Practice	1	7	12	**24**	44
9	Context	6	3	14	17	40
10	STEM	1	3	12	23	39
11	Instruction	3	4	12	17	36
12	Evidence	3	2	13	17	35
13	Cognition	4	2	9	14	29
14	Reasoning	0	3	8	14	25

*The bold font indicates the co-occurred times of more than the frequency average 23.5*.

The top 18 frequently co-occurring keywords of the articles published in the selected educational technology journals are presented in Table 5. The co-occurrence times of the keywords are more than the average number 23.5. The trend indicated a jump from the second period (2005–2009) to the third period (2010–2014). Scaffolding, support, and design were the top three frequently co-occurring keywords from the second period (2005–2009) to the fourth period (2015–2019). It was not until the second period (2005–2009) that the frequency of co-occurring keywords achieved 24. In addition to scaffolding (66), support (40), and design (33) related to scaffolding characteristics, cognition (26) related to learning performances, and STEM (24) related to learning disciplines were included in the third period (2010–2014). Compared with the same top three co-occurring keywords in the third period, three more co-occurring keywords were included: knowledge (29) related to learning performances, science education (28), and technology (25) related to scaffolding characteristics in the fourth period (2015–2019).

**Table 5:** The frequently co-occurred keywords in the selected educational technology journals.

**Rank**	**Keywords**	**2000–2004**	**2005–2009**	**2010–2014**	**2015–2019**	**Total**
1	Scaffolding	5	**24**	**66**	**67**	162
2	Support	1	12	**40**	**40**	93
3	Design	3	10	**33**	**44**	90
4	Knowledge	1	8	23	**29**	61
5	Cognition	2	10	**26**	20	58
6	Science education	0	8	20	**28**	56
7	Technology	1	8	21	**25**	55
8	Science	3	6	21	21	51
9	STEM	3	4	**24**	20	51
10	Learning environment	3	8	20	16	47
11	Teacher	1	1	21	20	43
12	Context	1	8	15	17	41
13	Interaction	1	4	17	17	39
14	Instruction	2	8	15	13	38
15	Practice	1	5	13	13	32
16	Computer	1	4	13	13	31
17	Assessment	1	3	11	15	30
18	Experiment	0	6	5	15	26

*The bold font indicates the co-occurred times of more than the frequency average 23.5*.

According to Tables 4, 5, six co-occurring keywords (technology, interaction, learning environment, computer, assessment, and experiment) were used in article published in science education journals. Two co-occurring keywords (evidence and reasoning) were absent from articles published in educational technology journals throughout the entire 20-year period.

### Results of Co-word Network Analysis

**Figure 5:**
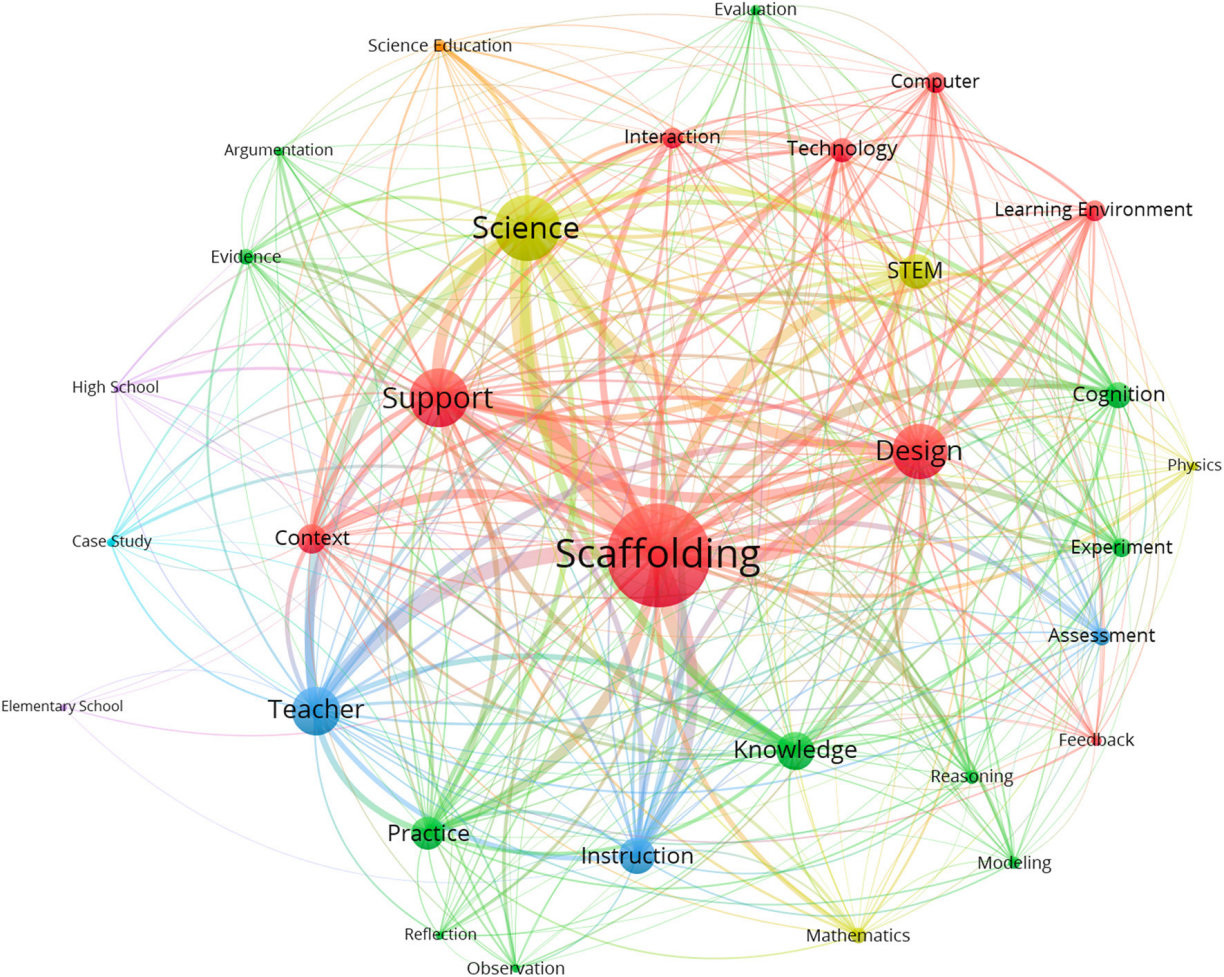
The co-occurred network of scaffolding research (All period: 2000–2019, keywords = 31).

To further uncover the trends of research themes in scaffolding research from 2000 to 2019, we conducted a series of CNA to depict the literature in the recent 20 years. Figure 5 shows the overview of the co-word analysis within the period of 2000–2019. It should be noted that the size of the node represents the frequency count of the keywords, while the thickness of the line indicates the strength of the co-occurrence of the keywords. The figure visualizes the aforementioned research themes regarding scaffolding that the researchers were interested in during the past decades.

With regard to the threshold number of frequently co-occurring keywords as 23.5, the co-occurrence frequencies found in the period of 2000–2004 were insufficient to conduct CNA. This implies that the mutual research interests regarding scaffolding in this period were still divergent. This may have hence led the articles to share relatively fewer co-words.

**Figure 6:**
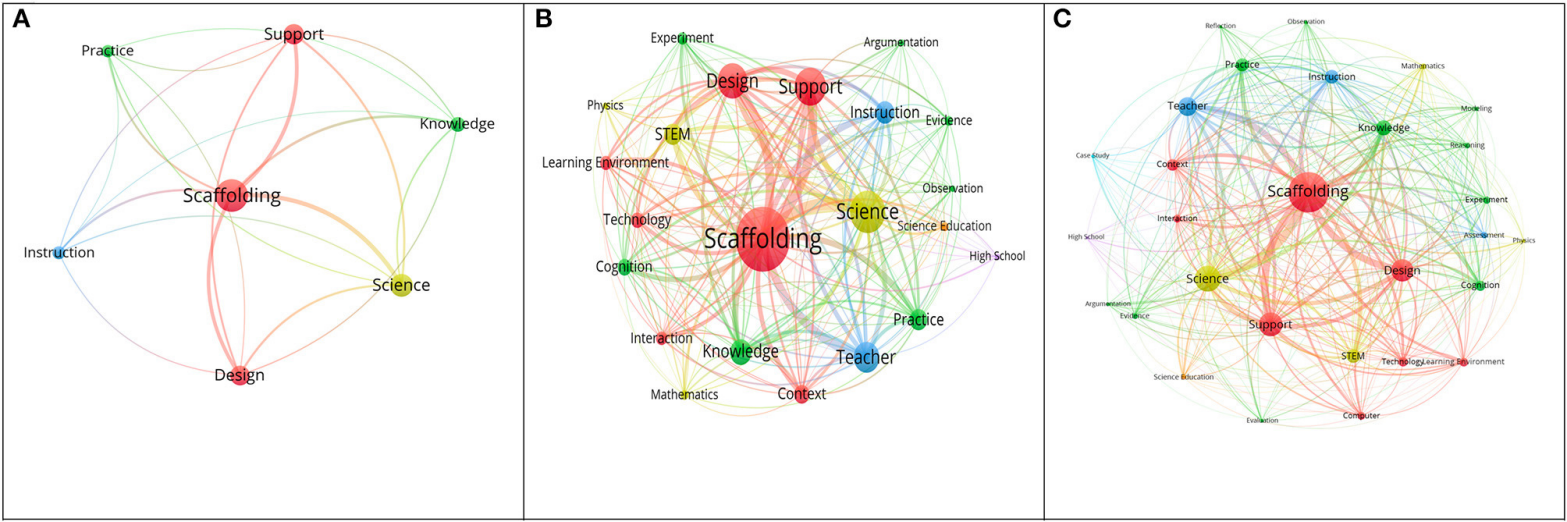
**(A)** The co-occurred network of scaffolding research (2005–2009, keywords = 7). **(B)** The co-occurred network of scaffolding research (2010–2014, keywords = 22). **(C)** The co-occurred network of scaffolding research (2015–2019, keywords = 29).

As for the period 2005–2009 (Figure 6A), seven keywords emerged in the co-occurrence network of the scaffolding research. Among these seven words, the links between “scaffolding and science” (the co-word frequency is 51), “scaffolding and design” (42), and “scaffolding and support” (41) are relatively higher than those between “scaffolding and knowledge” (36), “scaffolding and instruction” (32), and “scaffolding and practice” (27). Obviously, how to support students' science learning through designing proper scaffolding is one of the important issues in the relevant research field.

There were 22 keywords related to scaffolding which emerged from the co-word analysis of scaffolding research during 2010–2014, relatively much more than in 2005–2009 (Figure 6B). In addition to the higher frequency of the four co-words mentioned in 2005–2009 (scaffolding, science, support, and design), stronger links of the other co-words existed between the words “scaffolding and teacher” (93) and “scaffolding and instruction” (63), which belong to the research theme teaching; and “scaffolding and knowledge” (68), “scaffolding and practice” (53), and “scaffolding and cognition” (51), which belong to the research theme learning performances. Other stronger links of co-words also existed between “scaffolding and STEM” (65) and “scaffolding and context” (57). It is worth noting that the word “teacher” highly co-occurred with the word “scaffolding” compared to the other words. This might mean that the follow-up studies paid much more attention to teachers' roles in the design or use of scaffolding.

Compared with Figures 6A–C shows that more keywords (*N* = 29) and more complex links were identified in the co-word analysis of scaffolding research during 2015–2019. The four co-words (i.e., scaffolding, science, support, and design) still rank as the highest. The relationships among other highly co-occurring keywords mentioned in the period 2010–2014 were also enhanced. Especially, the co-words belonging to the research theme scaffolding characteristics, such as technology, computer, interaction, learning environment, and experiment increased greatly during this period. Some of the co-words belonging to the research theme learning performances, such as reasoning, evidence, argumentation, and evaluation, obviously appeared. It was revealed that in this period researchers seemed to focus more on the design of scaffolding enriched by technology or computers to improve students' higher order thinking skills. This also implies that researchers commonly appreciated the necessity to overcome teachers' difficulties to provide proper scaffoldings for supporting students' higher order thinking skills.

### Co-word Network Analysis Related to Participants

**Figure 7:**
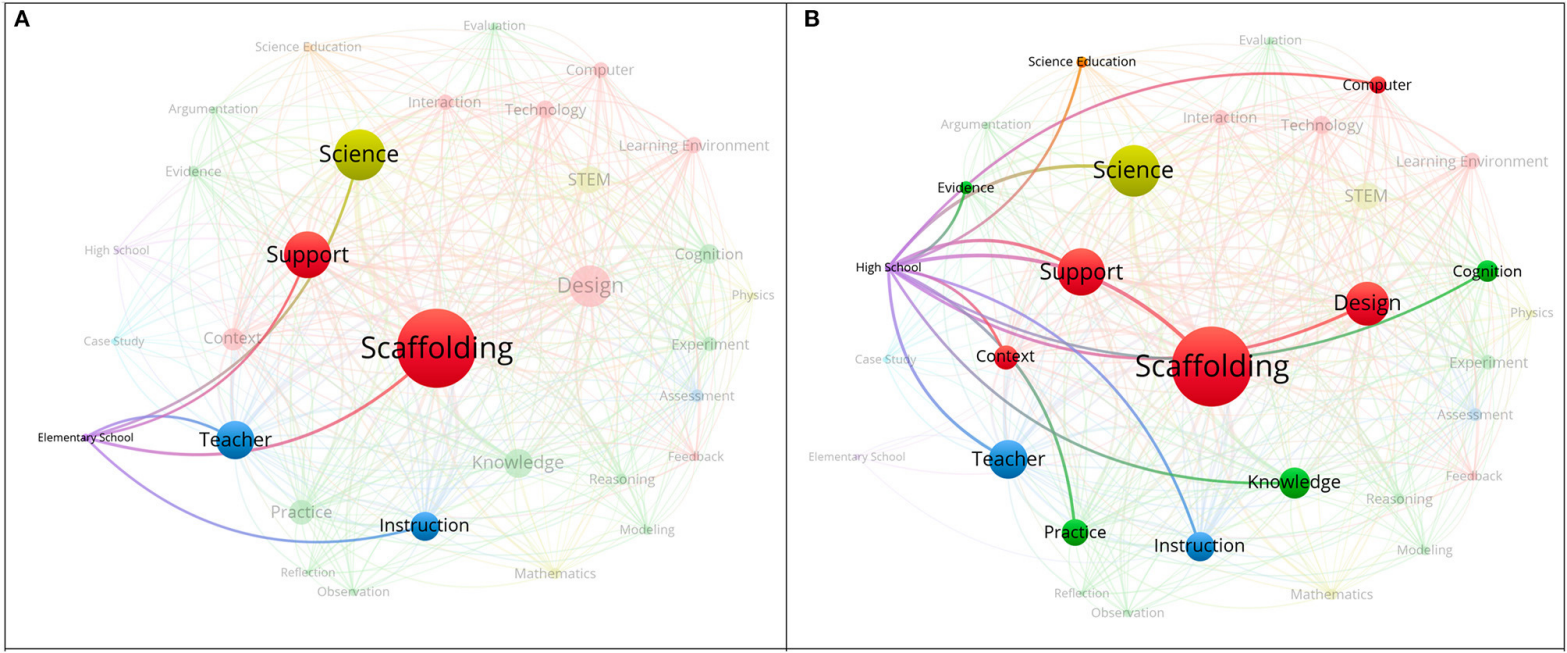
**(A)** “Elementary school” and other frequently co-word network analysis (keywords = 5). **(B)** “High school” and other frequently co-word network analysis (keywords = 13).

In the selected articles published in the recent two decades, there existed co-occurrence relationships among scaffolding, support, teacher, instruction, science, and elementary school (Figure 7A). Researchers who aimed at scaffolding elementary school learners may specifically tend to address the role of scaffolding characteristics, teaching, and the science discipline itself in their studies (Baker et al., 2008; Kershner et al., 2010; Choi et al., 2015; Decristan et al., 2015; van Uum et al., 2017). By contrast, more complicated co-word relationships are found in the network associated with scaffolding in high school context (Figure 7B). More keywords include scaffolding, support, design, context, and computer related to the research theme of scaffolding characteristics; teacher and instruction related to the research theme of teaching; knowledge, practice, cognition, and evidence related to the research theme of learning performances; science related to the research theme of learning disciplines; as well as science education. It seems that the researchers tended to specify scaffolding-related investigations with a more sophisticated manner while they focused on high school level participants. This also to some extent echoes Authors' study (2012) in that a major part of the literature regarding scaffolding gathered empirical data from students in high school contexts (Lin T. C. et al., 2012). Moreover, these researchers also paid attention to the application of computers in scaffolding design (Fang et al., 2016; Dasgupta et al., 2019; Tucker-Raymond et al., 2019; Zheng et al., 2019). The application of computers substantially elaborated the scaffolding that high school learners may require, and hence improved their learning performances in science (Marsteller and Bodzin, 2015; Kern and Crippen, 2017; Moser et al., 2017; Correia et al., 2019; Kyza and Georgiou, 2019).

### Co-word Network Analysis Related to Learning Performances

**Figure 8:**
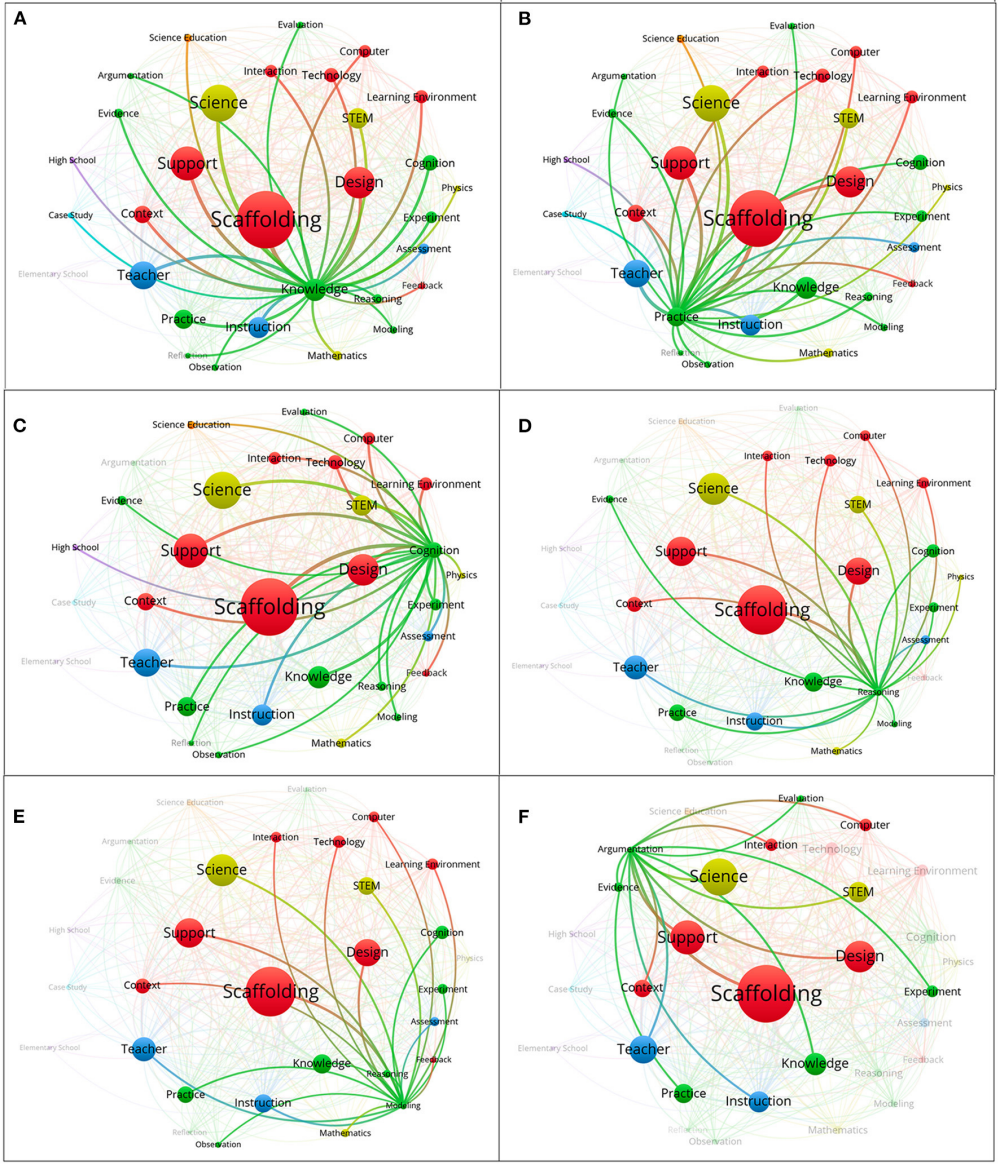
**(A)** “Knowledge” and other frequently co-word network analysis (keywords = 29). **(B)** “Practice” and other frequently co-word network analysis (keywords = 29). **(C)** “Cognition” and other frequently co-word network analysis (keywords = 27). **(D)** “Reasoning” and other frequently co-word network analysis (keywords = 21). **(E)** “Modeling” and other frequently co-word network analysis (keywords = 21). **(F)** “Argumentation” and other frequently co-word network analysis (keywords = 15).

Scaffolding is historically deemed as an effective procedure in instruction that provides learners with assistance or support to perform certain tasks regarding learning. The CNA results hence depict an outline of how researchers notably associated scaffold designs with learning performances (Figure 8A). It is anticipated that improvement in learning performance critically guaranteed researchers' meticulous arrangement of scaffolding in their investigations. Almost one-third of the selected articles in this study focused on the co-occurring word “knowledge.” This indicates that educators showed remarkable interest in guiding learners' growth of knowledge in light of improvement of academic achievement and conceptual learning. In Fund's (2007) study, the researcher identified effective components of scaffolding in a computerized environment that may positively contribute to junior high school students' learning outcomes. Chen et al. (2016) focused on scaffolding senior high school students' goal setting and planning in learning concepts regarding Boyle's law. This experimental study indicated that male low achievers especially benefitted from the scaffolds built by the researchers. Similar to low achievers, Villanueva et al. (2012) have appealed to science educators to focus their attention on students with special needs. The researchers suggested science writing heuristics as an effective approach that may scaffold students' inquiry, peer-assisted learning, and explanation.

Recently, it has been found that technologies potentially support students' learning and afford additional ways for teacher professional development. Some researchers have proposed a framework to utilize technology for scientific knowledge construction and transfer (Bitan-Friedlander et al., 2004), and understanding of NOS (nature of science) (Sandoval and Reiser, 2004). For example, Ibanez et al. (2016) utilized an image-based augmented reality simulator to scaffold students' science learning. Their findings indicated that proper supports in the secondary school physics curriculum significantly improved students' knowledge about electricity. A review paper that investigated the studies related to mobile applications (apps) and devices for science learning (Zydney and Warner, 2016) found that mobile apps shared some common design features, cited situated learning theory as the theoretical foundation, and measured scientific knowledge as the most common learning outcomes. Falloon (2017) explored how apps on mobile devices (e.g., iPads) supported hands-on science learning. He also discovered the limitations in the apps to support knowledge development, identified the critical roles of teachers, and clarified the importance of task structure in the promotion of students' conceptual development. Therefore, it is suggested that researchers need to align technologies and learning tools with instructional designs and learning theories for effective supports of students' learning.

Beyond the knowledge perspective, researchers have also tried to reveal possible contributions of scaffolding that benefit learners' learning practice (Fretz et al., 2002; Puente et al., 2013; Fang et al., 2016), cognition in learning (Magana et al., 2012; Zydney et al., 2014; López-Vargas et al., 2017) as well as reasoning (Silk et al., 2009; Kyza et al., 2011; Eggert et al., 2017) (Figures 8B–D). For example, Reilly's et al.'s study (2019) demonstrated how thinking scaffolds provided by teachers supported students' reasoning in technology-mediated problem solving and applying experimentation strategies to engage students in scientific reasoning in authentic contexts (Varma, 2014). Applying technological innovation to students' collaborative problem solving (Bressler et al., 2019) and reflection (Berglas-Shapiro et al., 2017) increased in the 5-year period of 2015–2019. As an example of exploring the connections among communications and scientific practices, researchers utilized a mobile game as a collaborative and scaffolding tool to foster collaborative problem solving (Bressler et al., 2019). It is noteworthy that these keywords were found to be overwhelmingly associated with all the key co-occurring words. Such findings hence echo previous reviews of science education literature about increasing academic publications that address learner characteristics (Lin et al., 2014, 2019) and assessing their higher order thinking performances (Zydney and Warner, 2016). There are still remaining research spaces in scaffolding students' development of cognition, reasoning, and scientific practices such as cognitive loads, assessment of higher order thinking performances, and the affordances of technology.

In addition, modeling is prevalent in the selected literature. Modeling is commonly deemed as a complicated mental activity in learning science. Such mental activity requires skills such as analysis, synthesis, and evaluation (VanLehn, 2013). It is not difficult to imagine that learners require certain scaffolds from knowledgeable guides while conducting models (Fretz et al., 2002; Wu et al., 2010; Corpuz and Rebello, 2019). Modeling a natural phenomenon is of value in science learning including explanation of empirical evidence and epistemological shifts when students construct and revise models with technological tools, the teacher's guidance, and peer discussions (Baek and Schwarz, 2015). However, the findings in this study show that the co-occurring relationships between modeling and evidence, modeling and evaluation, as well as modeling and reflection are unexpectedly handful (Figure 8E). The reasons for the absent co-occurrence of “modeling and evidence” and “modeling and evaluation” might be attributed to researchers' prevalent interests regarding students' modeling processes in scientific experiments or their mental representations of scientific models. According to the CNA results, studies that simultaneously aim at scaffolding and learners' higher order thinking skills may still require further research efforts.

Compared to the co-word network analysis of modeling, “argumentation” was frequently co-worded with evidence and evaluation, but fewer co-words with limited relationships were uncovered (Figure 8F). This reflects a situation contrary to the relevant research trend revealed in previous studies. Lin et al. (2014) and Lin et al. (2019) studies identified argumentation as a frequently published and highly cited issue in the field of science education within the most recent decade. However, while we examined the literature regarding scaffolding, only a handful of studies investigated argumentation at the same time. In the argumentation process, learners not only need the abilities of developing and evaluating arguments based on evidence, but also the ability of examining rebuttals in reflective ways. Moreover, interactivities and milieus in conventional classrooms are additional concerns in developing proper scaffolds for argumentation. To meet the possible difficulties, educators may further consider applying information technologies (Lin H. S. et al., 2012; Belland et al., 2016, 2019) and computer-based argumentation scaffolds (Fretz et al., 2002; Ravenscroft, 2007; Belland et al., 2011) to support learners' argumentation.

## Conclusions and Implications

The present study reviewed publications in influential literature from 2000 to 2019. Through CNA of the titles, abstracts, and standardized set of keywords of the selected articles, this study unveiled characteristics and trends regarding scaffolding within the recent 20 years. The number of scaffolding-relevant articles shows an obvious growth from 2005–2009 to 2010–2014. In line with Authors' (2012) study, the findings confirmed the increasing trend of scaffolding in both fields of science education and educational technology from various methodological perspectives (Lin T. C. et al., 2012).

Based on the findings from a total of 637 scaffolding-relevant papers, 286 author-defined keywords used by at least two studies were extracted. Among the identified keywords, “scaffolding,” “support,” and “design” were the top three most frequently used keywords during 2000 and 2019. We hence mapped and concluded six major groups of keywords applied in the selected articles to shape researchers' mutual interest while conducting research relevant to scaffolding. The six groups include scaffolding characteristics, learning performances, teaching, learning disciplines, grades of learners, as well as methodology. Visualization of co-word networks in each 5-year period during 2000 and 2019 revealed an obvious growth of researchers' efforts on “scaffolding characteristics” and “learning performances.” More complicated co-word relationships around the keyword “high school” also to some extent reflect both potential and difficulties researchers may encounter while conducting empirical investigations beyond high school contexts. This attempt to integrate co-word network analysis and visualization techniques provides insights regarding certain directions for consecutive investigations. Potentially existing research gaps and niches have also been presented and discussed.

Inevitably, this study may have its own limitations. Regarding the co-word analysis in this study, it is a dilemma to decide which part of each article should be dictionarized. Indeed, failing to dictionarize the full text of all articles may result in a certain degree of miscounting the co-word relationships. It is difficult to conjecture whether or not a word plays a critical role in the main text of the article. In other words, to dictionarize the whole article may strongly diminish the precision of the data analysis. We hence dictionarized only the article titles and abstracts based on the assumption that researchers are inclined to include the most relevant words in these two parts.

## Author Contributions

T-CL, S-SL, M-LC, and Y-SH contributed to the study conception and design. Material preparation, data collection, and analysis were performed by K-YT and T-CL. Y-SH supervised the research. The first draft of the manuscript was written by all authors. All authors have read and approved the final manuscript.

## Funding

This work was financially supported by the Institute for Research Excellence in Learning Sciences of National Taiwan Normal University (NTNU) from The Featured Areas Research Center Program within the framework of the Higher Education Sprout Project by the Ministry of Education (MOE) in Taiwan.

## Conflict of Interest

The authors declare that the research was conducted in the absence of any commercial or financial relationships that could be construed as a potential conflict of interest.

## Publisher's Note

All claims expressed in this article are solely those of the authors and do not necessarily represent those of their affiliated organizations, or those of the publisher, the editors and the reviewers. Any product that may be evaluated in this article, or claim that may be made by its manufacturer, is not guaranteed or endorsed by the publisher.

## References

[B1] Alrawili K. S. Osman K. Almuntasheri S. (2020). Effect of scaffolding strategies on higher-order thinking skills in science classroom. J. Baltic Sci. Educ. 19, 718–729. 10.33225/jbse/20.19.718

[B2] Assefa S. G. Rorissa A. (2013). A bibliometric mapping of the structure of STEM education using co-word analysis. J. Am. Soc. Inf. Sci. Technol. 64, 2513–2536. 10.1002/asi.2291725855820

[B3] Azevedo R. Cromley J. G. Seibert D. (2004). Does adaptive scaffolding facilitate students' ability to regulate their learning with hypermedia? Contemp. Educ. Psychol. 29, 344–370. 10.1016/j.cedpsych.2003.09.002

[B4] ^*^ Baek H. Schwarz C. V. (2015). The influence of curriculum, instruction, technology, and social interactions on two fifth-grade students' epistemologies in modeling throughout a model-based curriculum unit. J. Sci. Educ. Technol. 24, 216–233. 10.1007/s10956-014-9532-6

[B5] ^*^ Baker J. A. Clark T. P. Maier K. S. Viger S. (2008). The differential influence of instructional context on the academic engagement of students with behavior problems. Teach. Teach. Educ. 24, 1876–1883. 10.1016/j.tate.2008.02.019

[B6] ^*^ Belland B. R. Glazewski K. D. Richardson J. C. (2011). Problem-based learning and argumentation: testing a scaffolding framework to support middle school students' creation of evidence-based arguments. Instruct. Sci. 39, 667–694. 10.1007/s11251-010-9148-z

[B7] ^*^ Belland B. R. Gu J. Y. Kim N. J. Turner D. J. (2016). An ethnomethodological perspective on how middle school students addressed a water quality problem. Educ. Technol. Res. Dev. 64, 1135–1161. 10.1007/s11423-016-9451-8

[B8] ^*^ Belland B. R. Walker A. E. Kim N. J. (2017). Synthesizing results from empirical research on computer-based scaffolding in stem education: a meta-analysis. Rev. Educ. Res. 87, 309–344. 10.3102/0034654316670999 28344365 PMC5347356

[B9] ^*^ Belland B. R. Weiss D. M. Kim N. J. Piland J. Gu J. Y. (2019). An examination of credit recovery students' use of computer-based scaffolding in a problem-based, scientific inquiry unit. Int. J. Sci. Math. Educ. 17, 273–293. 10.1007/s10763-017-9872-9

[B10] ^*^ Berglas-Shapiro T. Eylon B. S. Scherz Z. (2017). A technology-enhanced intervention for self-regulated learning in science. Teach. Coll. Rec. 119, 1–26. 10.1177/016146811711901301

[B11] ^*^ Bitan-Friedlander N. Dreyfus A. Milgrom Z. (2004). Types of “teachers in training”: the reactions of primary school science teachers when confronted with the task of implementing an innovation. Teach. Teach. Educ. 20, 607–619. 10.1016/j.tate.2004.06.007

[B12] Bliss J (1994). “Children learning science,” in Wonder and Delight: Essays in Science Education in Honour of the Life and Work of Eric Rogers, eds. B. Jennison and J. Ogborn (Bristol: Institute of Physics Publishing), 45–61.

[B13] Bliss J. Askew M. Macrae S. (1996). Effective teaching and learning: scaffolding revisited. Oxford Rev. Educ. 22, 37–61. 10.1080/0305498960220103

[B14] ^*^ Bressler D. M. Bodzin A. M. Eagan B. Tabatabai S. (2019). Using epistemic network analysis to examine discourse and scientific practice during a collaborative game. J. Sci. Educ. Technol. 28, 553–566. 10.1007/s10956-019-09786-8

[B15] Brownfield K. Wilkinson I. A. G. (2018). Examining the impact of scaffolding on literacy learning: a critical examination of research and guidelines to advance inquiry. Int. J. Educ. Res. 90, 177–190. 10.1016/j.ijer.2018.01.004

[B16] Callon M. Courtial J. P. Laville F. (1991). Co-word analysis as a tool for describing the network of interactions between basic and technological research: the case of polymer chemistry. Scientometrics 22, 155–205. 10.1007/BF02019280

[B17] Cambrosio A. Limoges C. Courtial J. P. Laville F. (1993). Historical scientometrics? Mapping over 70 years of biological safety research with coword analysis. Scientometrics 27, 119–143. 10.1007/BF02016546

[B18] Chen C. K. Huang N. T. Hwang G. J. (in press). Findings implications of flipped science learning research: a review of journal publications. Interact. Learn. Environ. 10.1080/10494820.2019.1690528

[B19] ^*^ Chen S. F. Huang C. C. Chou T. L. (2016). The effect of metacognitive scaffolds on low achievers' laboratory learning. Int. J. Sci. Math. Educ. 14, 281–296. 10.1007/s10763-015-9691-9

[B20] ^*^ Choi A. Klein V. Hershberger S. (2015). Success, difficulty, and instructional strategy to enact an argument-based inquiry approach: experiences of elementary teachers. Int. J. Sci. Math. Educ. 13, 991–1011. 10.1007/s10763-014-9525-1

[B21] ^*^ Corpuz E. D. Rebello N. S. (2019). Refining students' explanations of an unfamiliar physical phenomenon-microscopic friction. Res. Sci. Educ. 49, 1177–1211. 10.1007/s11165-017-9650-2

[B22] ^*^ Correia A. P. Koehler N. Thompson A. Phye G. (2019). The application of PhET simulation to teach gas behavior on the submicroscopic level: secondary school students' perceptions. Res. Sci. Technol. Educ. 37, 193–217. 10.1080/02635143.2018.1487834

[B23] ^*^ Dasgupta C. Magana A. J. Vieira C. (2019). Investigating the affordances of a CAD enabled learning environment for promoting integrated STEM learning. Comput. Educ. 129, 122–142. 10.1016/j.compedu.2018.10.014

[B24] ^*^ Davis E. A. Linn M. C. (2000). Scaffolding students' knowledge integration: prompts for reflection in KIE. Int. J. Sci. Educ. 22, 819–837. 10.1080/095006900412293

[B25] ^*^ Decristan J. Hondrich A. L. Buttner G. Hertel S. Klieme E. Kunter M.. (2015). Impact of additional guidance in science education on primary students' conceptual understanding. J. Educ. Res. 108, 358–370. 10.1080/00220671.2014.899957

[B26] Dehdarirad T. Villarroya A. Barrios M. (2014). Research trends in gender differences in higher education and science: a co-word analysis. Scientometrics 101, 273–290. 10.1007/s11192-014-1327-2

[B27] ^*^ Eggert S. Nitsch A. Boone W. J. Nuckles M. Bogeholz S. (2017). Supporting students' learning and socioscientific reasoning about climate change-the effect of computer-based concept mapping scaffolds. Res. Sci. Educ. 47, 137–159. 10.1007/s11165-015-9493-7

[B28] ^*^ Falloon G (2017). Mobile devices and apps as scaffolds to science learning in the primary classroom. J. Sci. Educ. Technol. 26, 613–628. 10.1007/s10956-017-9702-4

[B29] Fang S. C. Hsu Y. S. Hsu W. H. (2016). Effects of explicit and implicit prompts on students' inquiry practices in computer-supported learning environments in high school earth science. Int. J. Sci. Educ. 38, 1699–1726. 10.1080/09500693.2016.1213458

[B30] ^*^ Fretz E. B. Wu H. K. Zhang B. H. Davis E. A. Krajcik J. S. Soloway E. (2002). An investigation of software scaffolds supporting modeling practices. Res. Sci. Educ. 32, 567–589. 10.1023/A:1022400817926

[B31] ^*^ Fund Z (2007). The effects of scaffolded computerized science problem-solving on achievement outcomes: a comparative study of support programs. J. Comput. Assist. Learn. 23, 410–424. 10.1111/j.1365-2729.2007.00226.x

[B32] Hsu Y. S. Lai T. L. Hsu W. H. (2015). A design model of distributed scaffolding for inquiry-based learning. Res. Sci. Educ. 45, 241–273. 10.1007/s11165-014-9421-2

[B33] Huang C. Yang C. Wang S. Wu W. Su J. Liang C. (2020). Evolution of topics in education research: a systematic review using bibliometric analysis. Educ. Rev. 72, 281–297. 10.1080/00131911.2019.156621232152856

[B34] ^*^ Ibanez M. B. Di-Serio A. Villaran-Molina D. Delgado-Kloos C. (2016). Support for agmented reality simulation systems: the effects of scaffolding on learning outcomes and behavior patterns. IEEE Trans. Learn. Technol. 9, 46–56. 10.1109/TLT.2015.244576127295638

[B35] Kelley T. L (1927). Interpretation of Educational Measurements. New York, NY: Macmillan.

[B36] ^*^ Kern C. L. Crippen K. J. (2017). The effect of scaffolding strategies for inscriptions and argumentation in a science cyberlearning environment. J. Sci. Educ. Technol. 26, 33–43. 10.1007/s10956-016-9649-x

[B37] ^*^ Kershner R. Mercer N. Warwick P. Staarman J. K. (2010). Can the interactive whiteboard support young children's collaborative communication and thinking in classroom science activities? Int. J. Comput. Support. Collab. Learn. 5, 359–383. 10.1007/s11412-010-9096-2

[B38] ^*^ Kim M. C. Hannafin M. J. (2011). Scaffolding problem solving in technology-enhanced learning environments (TELEs): bridging research and theory with practice. Comput. Educ. 56, 403–417. 10.1016/j.compedu.2010.08.024

[B39] ^*^ Kyza E. A. Constantinou C. P. Spanoudis G. (2011). Sixth graders' co-construction of explanations of a disturbance in an ecosystem: exploring relationships between grouping, reflective scaffolding, and evidence-based explanations. Int. J. Sci. Educ. 33, 2489–2525. 10.1080/09500693.2010.550951

[B40] ^*^ Kyza E. A. Georgiou Y. (2019). Scaffolding augmented reality inquiry learning: the design and investigation of the TraceReaders location-based, augmented reality platform. Interact. Learn. Environ. 27, 211–225. 10.1080/10494820.2018.1458039

[B41] ^*^ Land S. M. Zembal-Saul C. (2003). Scaffolding reflection and articulation of scientific explanations in a data-rich, project-based learning environment: an investigation of progress portfolio. Educ. Technol. Res. Dev. 51, 65–84. 10.1007/BF02504544

[B42] Leydesdorff L (1989). The relations between qualitative theory and scientometric methods in science and technology studies. Scientometrics 15, 333–347. 10.1007/BF02017058

[B43] ^*^ Lin H. S. Hong Z. R. Lawrenz F. (2012). Promoting and scaffolding argumentation through reflective asynchronous discussions. Comput. Educ. 59, 378–384. 10.1016/j.compedu.2012.01.019

[B44] ^*^ Lin T. C. Hsu Y. S. Lin S. S. Changlai M. L. Yang K. Y. Lai T. L. (2012). A review of empirical evidence on scaffolding for science education. Int. J. Sci. Math. Educ. 10, 437–455. 10.1007/s10763-011-9322-z24033240

[B45] Lin T. C. Lin T. J. Tsai C. C. (2014). Research trends in science education from 2008 to 2012: A systematic content analysis of publications in selected journals. Int. J. Sci. Educ. 36, 1346–1372. 10.1080/09500693.2013.864428

[B46] Lin T. J. Lin T. C. Potvin P. Tsai C. C. (2019). Research trends in science education from 2013 to 2017: A systematic content analysis of publications in selected journals. Int. J. Sci. Educ. 41, 367–387. 10.1080/09500693.2018.1550274

[B47] López-Belmonte J. Moreno-Guerrero A. J. Pozo-Sánchez S. Marín-Marín J. A. (2021). Co-word analysis and academic performance from the Australasian Journal of Educational Technology in Web of Science. Aust. J. Educ. Technol. 37, 119–140. 10.14742/ajet.6940

[B48] ^*^ López-Vargas O. Ibáñez-Ibáñez J. Racines-Prada O. (2017). Students' metacognition and cognitive style and their effect on cognitive load and learning achievement. Educ. Technol. Soc. 20, 145–137. 10.1177/1365480217704263

[B49] ^*^ Magana A. J. Brophy S. P. Bryan L. A. (2012). An integrated knowledge framework to characterize and scaffold size and scale cognition (FS2C). Int. J. Sci. Educ. 34, 2181–2203. 10.1080/09500693.2012.715316

[B50] Marín-Marín J. A. Moreno-Guerrero A. J. Dúo-Terrón P. López-Belmonte J. (2021). STEAM in education: a bibliometric analysis of performance and co-words in Web of Science. Int. J. STEM Educ. 8, 41. 10.1186/s40594-021-00296-x 34189015 PMC8226355

[B51] ^*^ Marsteller R. B. Bodzin A. M. (2015). The effectiveness of an online curriculum on high school students' understanding of biological evolution. J. Sci. Educ. Technol. 24, 803–817. 10.1007/s10956-015-9565-5

[B52] ^*^ McNeill K. L. Lizotte D. J. Krajcik J. Marx R. W. (2006). Supporting students' construction of scientific explanations by fading scaffolds in instructional materials. J. Learn. Sci. 15, 153–191. 10.1207/s15327809jls1502_1

[B53] Metz K. E (1995). Reassessment of developmental constraints on children's science instruction. Rev. Educ. Res. 65, 93–127. 10.3102/00346543065002093

[B54] Metz K. E (1997). On the complex relation between cognitive developmental research and children's science curricula. Rev. Educ. Res. 67, 151–163. 10.3102/00346543067001151

[B55] ^*^ Moser S. Zumbach J. Deibl I. (2017). The effect of metacognitive training and prompting on learning success in simulation-based physics learning. Sci. Educ. 101, 944–967. 10.1002/sce.2129525855820

[B56] Noroozi O. Kirschner P. A. Biemans H. J. Mulder M. (2018). Promoting argumentation competence: extending from first- to second-order scaffolding through adaptive fading. Educ. Psychol. Rev. 30, 153–176. 10.1007/s10648-017-9400-z

[B57] Oh S. Jonassen D. H. (2007). Scaffolding online argumentation during problem solving. J. Comput. Assist. Learn. 23, 95–110. 10.1111/j.1365-2729.2006.00206.x

[B58] ^*^ Puente S. M. G. van Eijck M. Jochems W. (2013). A sampled literature review of design-based learning approaches: a search for key characteristics. Int. J. Technol. Des. Educ. 23, 717–732. 10.1007/s10798-012-9212-x

[B59] ^*^ Quintana C. Reiser B. J. Davis E. A. Krajcik J. Fretz E. Duncan R. G.. (2004). A scaffolding design framework for software to support science inquiry. J. Learn. Sci. 13, 337–386. 10.1207/s15327809jls1303_4

[B60] ^*^ Ravenscroft A (2007). Promoting thinking and conceptual change with digital dialogue games. J. Comput. Assist. Learn. 23, 453–465. 10.1111/j.1365-2729.2007.00232.x

[B61] ^*^ Reilly C. M. Kang S. Y. Grotzer T. A. Joyal J. A. Oriol N. E. (2019). Pedagogical moves and student thinking in technology-mediated medical problem-based learning: supporting novice-expert shift. Br. J. Educ. Technol. 50, 2234–2250. 10.1111/bjet.12843

[B62] ^*^ Reiser B. J (2004). Scaffolding complex learning: the mechanisms of structuring and problematizing student work. J. Learn. Sci. 13, 273–304. 10.1207/s15327809jls1303_2

[B63] Rogoff B (1990). Apprenticeship in Thinking: Cognitive Development in Social Context. Oxford: Oxford University Press.

[B64] Rubio D. M. Berg-Weger M. Tebb S. S. Lee E. S. Rauch S. (2003). Objectifying content validity: Conducting a content validity study in social work research. Soc. Work Res. 27, 94–104. 10.1093/swr/27.2.94

[B65] ^*^ Sandoval W. A. Reiser B. J. (2004). Explanation-driven inquiry: integrating conceptual and epistemic scaffolds for scientific inquiry. Sci. Educ. 88, 345–372. 10.1002/sce.1013025855820

[B66] ^*^ Silk E. M. Schunn C. D. Cary M. S. (2009). The impact of an engineering design curriculum on science reasoning in an urban setting. J. Sci. Educ. Technol. 18, 209–223. 10.1007/s10956-009-9144-8

[B67] Tang K. T. Wang C. Y. Chang H. Y. Chen S. F. Lo H. C. Tsai C. C. (2016). The intellectual structure of metacognitive scaffolding in science education: A co-citation network analysis. Int. J. Sci. Math. Educ. 14, 249–262. 10.1007/s10763-015-9696-4

[B68] Toleda S. Dubas J. M. (2016). Encouraging higher-order thinking in general chemistry by scaffolding student learning using Marzano's taxonomy. J. Chem. Educ. 93, 64–69. 10.1021/acs.jchemed.5b00184

[B69] ^*^ Tucker-Raymond E. Puttick G. Cassidy M. Harteveld C. Troiano G. M. (2019). “I Broke Your Game!”: critique among middle schoolers designing computer games about climate change. Int. J. STEM Educ. 6:41. 10.1186/s40594-019-0194-z

[B70] ^*^ van Uum M. S. J. Verhoeff R. P. Peeters M. (2017). Inquiry-based science education: Scaffolding pupils' self-directed learning in open inquiry. Int. J. Sci. Educ. 39, 2461–2481. 10.1080/09500693.2017.1388940

[B71] ^*^ VanLehn K (2013). Model construction as a learning activity: a design space and review. Interact. Learn. Environ. 21, 371–413. 10.1080/10494820.2013.803125

[B72] ^*^ Varma K (2014). Supporting scientific experimentation and reasoning in young elementary school students. J. Sci. Educ. Technol. 23, 381–397. 10.1007/s10956-013-9470-8

[B73] ^*^ Villanueva M. G. Taylor J. Therrien W. Hand B. (2012). Science education for students with special needs. Stud. Sci. Educ. 48, 187–215. 10.1080/14703297.2012.737117

[B74] Vygotsky L. S (1978). Mind in Society: The Development of Higher Psychological Processes. Cambridge, MA: Harvard University Press.

[B75] Wood D. Bruner J. S. Ross G. (1976). The role of tutoring in problem solving. J. Child Psychol. Psychiatry Allied Discipl. 17, 89–100. 10.1111/j.1469-7610.1976.tb00381.x 932126

[B76] Wu H. K. Hsu Y. S. Hwang F. K. (2010). Designing a technology-enhanced learning environment to support scientific modeling. Turkish Online J. Educ. Technol. 9, 58–65.

[B77] Wu S. H. Lai C. L. Hwang G. J. Tsai C. C. (2021). Research trends in technology-enhanced chemistry learning: a review of comparative research from 2010 to 2019. J. Sci. Educ. Technol. 30, 496–510. 10.1007/s10956-020-09894-w

[B78] ^*^ Zheng J. Xing W. Zhu G. X. (2019). Examining sequential patterns of self- and socially shared regulation of STEM learning in a CSCL environment. Comput. Educ. 136, 34–48. 10.1016/j.compedu.2019.03.005

[B79] Zhou M. Lam K. K. (2019). Metacognitive scaffolding for online information search in K-12 and higher education settings: a systematic review. Educ. Technol. Res. Dev. 67, 1353–1384. 10.1007/s11423-019-09646-7

[B80] ^*^ Zydney J. M. Bathke A. Hasselbring T. S. (2014). Finding the optimal guidance for enhancing anchored instruction. Interact. Learn. Environ. 22, 668–683. 10.1080/10494820.2012.745436

[B81] ^*^ Zydney J. M. Warner Z. (2016). Mobile apps for science learning: review of research. Comput. Educ. 94, 1–17. 10.1016/j.compedu.2015.11.001

